# Genetic Analysis of Pathways to Parkinson Disease

**DOI:** 10.1016/j.neuron.2010.10.014

**Published:** 2010-10-21

**Authors:** John Hardy

**Affiliations:** 1 Reta Lilla Weston Laboratories and Department of Molecular Neuroscience, UCL Institute of Neurology, London WC1N 3BG, UK

## Abstract

In this review I outline the arguments as to whether we should consider Parkinson disease one or more than one entity and discuss genetic findings from Mendelian and whole-genome association analysis in that context. I discuss what the demonstration of disease spread implies for our analysis of the genetic and epidemiologic risk factors for disease and outline the surprising fact that we now have genetically identified on the order of half our risk for developing the disease.

## Main Text

We have two goals in the genetic analysis of disease: the first is to increase the accuracy of risk prediction, with an intention of getting better at diagnosing the disease earlier and perhaps targeting presymptomatic therapy; the second is to define the pathways that lead to cell death so that we can design approaches that intervene in those pathways and act as mechanistic therapies.

In Parkinson disease, a neurodegenerative disorder affecting movement and usually characterized by the presence of α-synuclein containing Lewy bodies in damaged neurons, genetic analysis has been remarkably successful, with many mendelian loci already described, a common high-risk variant identified and many common low-risk variants recently elucidated. This is particularly remarkable because epidemiologic investigations preceding the identification of the α-synuclein (SNCA) locus consistently suggested that genes were unimportant in disease etiology. As our knowledge of the disease has increased over the last 15 years, the proportion of risk assigned to the environment has consistently decreased and, while associations with environmental factors have been confirmed (such as that showing a negative association with smoking), no environmental risk factor with a convincing pathogenic role in the disease has yet been described.

We and others have recently comprehensively reviewed the identification of mendelian genes for Parkinson disease (Hardy et al., 2009; Klein and Lohmann-Hedrich, 2007; Cookson and Bandmann, 2010). Given this, my purpose in this review is to discuss four issues:

1. To discuss whether all the identified genes relate to single entity and what pathways have been identified as relevant to disease.
2. To discuss how the identification of risk loci through genome-wide association studies relate to the Mendelian genes and whether we should inevitably expect those low-risk loci to encode proteins that map onto the same pathways as those identified through the identification of Mendelian loci:
3. To discuss the implication of permissive templating (“prionoid”) behavior of α-synuclein to the concept of disease risk
4. To discuss the proportion of risk for disease that has been identified so far.

### Mendelian and High-Risk Loci for Parkinson Disease

Table 1 lists the loci at which pathogenic mutations lead to parkinsonism (for references, see Hardy et al., 2009). These include those loci traditionally noted as “Parkinson loci” as well as others that are not, including MAPT, SCA2, SCA3, and spastacsin, which can clinically present as Parkinson disease but often is clinically distinct. In some diseases, most clearly Alzheimer disease, the disease is defined by its pathology. In Parkinson disease, the disease has been traditionally defined clinically, but the vast majority of cases of disease have the pathology of Lewy bodies (Hughes et al., 1993). However, while the vast majority of idiopathic cases have Lewy bodies, the genetic forms of the disease have variable pathologies (Table 1). Thus part of my intention in this review is to question how we should group the genetic loci if we are trying to understand the pathogenesis of the disorder. Should we use clinical criteria (in which case, all of the loci may stake a claim as to involvement), or should we use pathological criteria, in which case the number clearly involved is much smaller although for many, the pathology has not been documented?

#### In Favor of Using Pathological Criteria

The major argument in favor of using pathological criteria for the definition of disease is an analogy: in Alzheimer disease, the loci identified for the disorder, APP, and the presenilins clearly map into one pathway (Hardy and Selkoe, 2002): defining the disease clinically would lead to confusion with a large series of other genes (MAPT, PRNP, PGRN, etc.) being grouped with them. A second argument is that both clinically and pathologically the disease seems to spread along neuronal pathway. This notion of the disease spreading has been most vividly captured by the work of Braak and colleagues (Braak et al., 2003) and is supported by the idea that misfolded synuclein acts as a template for other synuclein to deposit upon (see below). It is difficult to see how this templating pathogenesis could be relevant to those forms of the disease without Lewy body pathology.

#### Against Using Pathological Criteria: for Using Clinical Criteria

There are several arguments against using pathological criteria. First, the pathology of (for example) LRRK2 mutation carriers is variable with the majority of cases having Lewy bodies, but a minority have tangles and some having other pathology (Zimprich et al., 2004). Second, a few compound heterozygote parkin mutation cases (e.g., Farrer et al., 2010) and some clearly simple heterozygous parkin and some, at least, PINK1 cases have been reported to have typical Lewy body disease suggesting that the pathogeneses of the two forms of the disease are not dissimilar (Samaranch et al., 2010). Third, although mutations in MAPT (microtubule-associated protein tau, or often referred to simply as tau) lead to a disorder that can closely clinically resemble idiopathic Parkinson disease, the majority have a dementing syndrome that is clearly different from Parkinson disease; however, the MAPT/tau haplotype shows an association with disease (see below) strongly suggesting that the pathogenic cascades in the tauopathies must be related to those in the synucleinopathies.

A practical argument against using pathological criteria is that for many syndromes, we do not know the underlying pathology of many of the syndromes, and a lesson to be drawn from the case of LRRK2 mutations is that pathology can be variable.

#### Against Using Clinical Criteria

As Table 1 illustrates, there are many syndromes that can masquerade as Parkinson disease, some of which (e.g., SCA2 and SCA3) almost certainly have different pathogenic mechanisms since they are both polyglutamine repeat disorders. Furthermore, there are clear phenotypic differences between the diseases defined by their genetic etiology. As an example, the Japanese clinicians who first identified PARK2 encoded disease were clear that this was distinct from typical Parkinson disease, with a very prolonged and benign disease duration, profound dopamine sensitivity, and sleep benefit (Yamamura 2010); PINK1 and possibly (Bonifati et al., 2005) DJ-1 and FBXO7 encoded diseases seem similar (Paisán-Ruiz et al., 2010). This is clearly clinically different from typical idiopathic Parkinson disease (see Hughes et al., 1993)

#### What Pathways Have Come out of the Analysis of Mendelian Genes?

The pathway that has come most clearly out of the analysis of the Mendelian genes is a mitochondrial damage repair pathway. It is clear that parkin, an E3 ubiquitin ligase, and PINK1, a mitochondrial kinase, are genetically in the same pathway (with parkin downstream of PINK1) and that this pathway is involved in the elimination of damaged mitochondria (Park et al., 2006; Clark et al., 2006). DJ-1, and possibly FBX07, another ubiquitin ligase, are also likely to be involved in mitochondrial physiology. DJ-1 has been shown to translocate to the mitochondria and exert a protective effect on the application of oxidative stress (Canet-Avilés et al., 2004: see Cookson 2010). Whether the products of these genes are also directly involved in the same or different mitochondrial repair/elimination pathways is not yet clear. But the likelihood that all these genes map onto mitochondrial pathways and all have rather similar clinical phenotypes is striking (Paisán-Ruiz et al., 2010; Valente et al., 2004; Yamamura 2010). It is notable that MPTP toxicity is also both dopamine selective and mitochondrial (Langston 1989). Of course, MPTP selectivity is largely explicable through dopamine metabolism, but the clear impression is that dopamine and possibly other catecholamine neurons are selectively sensitive to mitochondrial damage (Schapira 2008; Dodson and Guo, 2007; Cookson and Bandmann, 2010).

A second pathway that is likely to be involved in Parkinson disease clearly involves the lysosomes. Glucosecerebrosidase and ATP13A2 are lysosomal enzymes (though the precise function of the latter is not yet clear). While there are interactions between lysosomal and mitochondrial metabolism (as there are between any cellular compartments), whether there is or should expected to be any close relationship between these loci and the mitochondrial loci is not clear.

The two loci that all would agree are central to Parkinson disease, SNCA and LRRK2, are, in many ways, the most mysterious. α-synuclein clearly has a role in synaptic release (Cabin et al., 2002) and is related to the SNARE complex proteins (Burré et al., 2010), and, as a kinase, LRRK2 is almost certainly involved in signaling cascades (Dauer and Ho, 2010), probably relating to cytoskeletal dynamics (MacLeod et al., 2006; Meixner et al., 2010). While there is some evidence that LRRK2 and SNCA may have a role in the same pathway (Lin et al., 2009), they do not appear to interact directly and their roles have no obvious connection to either lysosomal or mitochondrial metabolism.

### Low-Risk Loci for Parkinson Disease

Genome-wide association studies (GWAS) have revolutionized our efforts to find loci at which common, normal genetic variability contributes to disease risk. Since these findings, for all diseases, are relatively new, we have little experience in conceptualizing what this common variability is and how it fits into the jigsaw of pathogenesis. In general, most of the loci found by this strategy are present in more than 5% of the population (allele frequencies of > 10%) and, if you have the risk allele, increase your risk of disease less than two fold over the population average (have odds ratios OR < 2). As a comparator, possessing a single APOE4 allele (present in 10% of the population) increases your risk of Alzheimer disease about four-fold. The majority of these low-risk loci for all diseases seem to mediate their effect by altering gene expression rather than through protein coding changes. In fact, there is an approximate relationship that as the odds ratios at a locus increases the more likely that the encoding gene will have a protein coding alteration at its heart (Singleton et al., 2010).

In Parkinson disease GWAS, the fact that the SNCA locus was the first to reach genome-wide significance is perhaps not surprising (Simón-Sánchez et al., 2009) (since it had previously been suggested on the basis of candidate gene analysis: Krüger et al., 1999) and this is consistent with the general suggestion that genetic variability at the loci encoding deposited protein influences the risk of disease (Singleton et al., 2004). For instance, theMAPT locus is the most prominent locus for the tangle diseases and the prion locus the most prominent locus for Creutzfeldt-Jakob disease. Likewise, gene duplications at SNCA cause familial Parkinson disease: gene duplications at MAPT cause frontotemporal dementia and gene duplications at APP cause Alzheimer disease (for references, see Singleton et al., 2004). In the case of the SNCA locus, it seems likely that those in the population who express about 10% more than the average have an increase of developing Parkinson disease increased by about 40% (Simón-Sánchez et al., 2009; Fuchs et al., 2008). While this finding is intellectually satisfying, it does not radically alter our view of the pathogenesis of disease, although it does make it genetically clear that α-synuclein is central to the pathogenesis in idiopathic disease.

The fact that MAPT is the second locus to come out of the GWAS is more surprising, although it too had been previously identified as a candidate gene (Golbe et al., 2010). While tau pathology is sometimes found in Parkinson disease, it is not pathognomic. Furthermore the haplotype that is associated with Parkinson disease is distinct from that associated with the tangle disorder, progressive supranuclear palsy (Vandrovcova et al., 2009). Indeed, strictly, we cannot be sure that the association is with tau since the large haplotype that is associated with Parkinson disease has many other genes upon it (Fung et al., 2005). Finally, although we do not understand the relationship between the MAPT locus and Parkinson disease, it is worth remembering that while LRRK2 mutations usually give rise to α-synuclein pathology, they sometimes give rise to tangle pathology (Zimprich et al., 2004) and that, while APP mutations usually give rise to tangle pathology, they sometimes give rise to Lewy body pathology (Hardy 1994). In other words, there are at least three examples where there are genetic pathologic connections between α-synuclein and tau.

The association between LRRK2 and sporadic disease seen in GWAS is not yet understood. It is not clear whether the association is driven by genetic variability in the expression of LRRK2 (which would imply that normal function of LRRK2 was important in disease etiology) or whether the effect is driven by some of the relatively common pathogenic mutations among the samples that have been used in the studies. LRRK2 is a very large gene and by no means had all of the samples used in the studies been sequenced. Thus, we cannot yet tell whether the LRRK2 finding in GWAS represents a new finding or a rediscovery of an old one.

The identification of the HLA locus as reaching genome-wide significance is of great interest (Hamza et al., 2010). It too had been previously identified as a candidate locus for Parkinson disease (Saiki et al., 2010), but its recognition as an important locus for disease has profound implications both narrowly for Parkinson disease and more generally for our interpretation of the identification of risk loci for late-onset neurodegenerative disease. Similar to the recent GWAS pointing to the complement cascade as contributing to risk of Alzheimer disease (Harold et al., 2009; Lambert et al., 2009) and the identification of complement H as the major risk locus for macular degeneration (Klein et al., 2005), it points to the potential importance of genetic variability in damage repair and clean up as influencing risk for disease. In the case of Alzheimer disease and macular degeneration, as well as Parkinson disease, the adaptive or innate immune systems had previously been implicated in disease pathology (McGeer et al., 1988; McGeer et al., 1989; McGeer et al., 2005), though the precise role has not been clear. From these findings, one might propose that, given a pathogenic insult, at least some of these low-risk loci may not actually be important for disease etiology, either in triggering disease or disease progression or even in the pathways that initiate cell death, but rather are involved in the clearing out of the detritus. If this turns out to be the case, it is interesting to note that in the different diseases, different aspects of the immune system appear to play more or less important roles.

### The Importance of Permissive Templating—“Prionoid” Behavior—in the Pathogenesis of Parkinson Disease

Li and Kordower and their colleagues showed, in autopsy studies of patients with Parkinson disease who had, clinically successfully, received fetal dopmainergic grafts and survived more than 10 years had developed Lewy bodies in those grafts. This remarkable demonstration that grafted embryonic dopaminergic cells develop Lewy bodies after ∼10 years in the brain of someone with Parkinson disease (Li et al., 2008; Kordower et al., 2008) can be interpreted in two ways: first, that the environment the cells are in is conducive to Lewy body formation in some nonspecific way, or, alternatively, that Lewy body material from the surrounding tissue has templated the pathology in the graft neuron (Brundin et al., 2008). The latter interpretation is favored by analogy (Hardy 2005), not only with prion diseases, but also by analogy with experimental work on both Aβ and tau pathology spread (Meyer-Luehmann et al., 2006; Clavaguera et al., 2009; Frost and Diamond, 2010). In addition, direct cell to cell spread of α-synuclein aggregation has been demonstrated in vitro. These findings are, of course, completely consistent with the view of these diseases propounded by Braak and colleagues (Braak and Braak, 1998; Braak et al., 2003) who has proposed that both Alzheimer and Parkinson disease spread within the brain in a fairly consistent manner

These observations about how AD and PD pathology spread within the brain, across regions, imply that disease initiation need only to occur at a single site. If initiation need only occur once, then while factors that increase its likelihood will be risk factors (such as an increase in the concentration of its constituent protein), its precise initiation will be stochastic and not predictable and, therefore, may be due to stochastic events rather than predictable ones. The clear corollary of this suggestion is it may not be possible to completely predict who will get Parkinson disease (or the other diseases for which this is true): identical twins who live identical lives may have different outcomes vis-à-vis Parkinson disease because of stochastic initiation events.

### How Much Risk Has Been Found?

As we continue our genetic analysis, we are finding more and more alleles at several genes that increase our risk of disease. One important question is how much of the risk for disease have we found? If we could take all the risk alleles for disease out of circulation, how much would we reduce the incidence of the disease? The amount of risk for Parkinson disease we have found varies enormously from population to population and the amount accounted for by different risk loci also varies across populations. Among the Ashkenazim, a very large proportion of risk, perhaps 40%, is accounted for by LRRK2 (G2019S) and GBA mutations (Ozelius et al., 2006; Sidransky et al., 2009); in Arab populations too, LRRK2 G2019S is an important factor (Lesage et al., 2006). In East Asian populations, there are a number of common LRRK2 variants that considerably alter risk (Mata et al., 2005; Ross et al., 2008). Together, coding variability at LRRK2 explains about 10% of risk in these populations (Tan et al., 2010). In outbred European populations, the proportion of risk due to coding variability in LRRK2 and GBA is less but still appreciable at about 8% (Gilks et al., 2005; Sidransky et al., 2009). Parkin and to a lesser extent PINK1 mutations are common in early-onset disease and may explain about 50% of the disease with an age at onset under 40: but this is probably only of the order of 1%–2% of the disease overall (Ibáñez et al., 2006). Thus, Mendelian and identified high-risk loci explain between ∼10% and ∼40% of risk in most populations so far assessed.

Working out the proportion of risk encoded by common low-risk loci is less straightforward, but it seems that in European populations, the SNCA, MAPT, and HLA loci each explain of the order of 10% of risk (Simón-Sánchez et al., 2009; Hamza et al., 2010). These figures are, of course, derived from mainly clinic-based series of cases and are not population based, an approach that generally leads to an overestimate of the amount of risk that has been identified. However, these risk calculations also only take into account the most common variant identified at each locus, and, undoubtedly, further analysis will identify other risk variants at each locus.

While all of these calculations are, of necessity, back-of-envelope calculations, they imply that genetic analysis has already identified of the order of half of the attributable risk of getting Parkinson disease. As more loci are discovered and as more analysis is done at each identified locus, this proportion of identified risk will clearly increase substantially.

### Conclusions

The 15 years since the identification of the SNCA locus in the Contursi kindred have seen enormous progress in Parkinson research to the extent where we now have identified about half of the risk of developing the disease. What now are our challenges? Clearly, finding other genes involved in the disease will, like finding pieces of a jigsaw, help us more clearly see the whole picture. We need to work out whether all the genes I have mentioned fit into one puzzle or whether there are two or more puzzles. α-synuclein and LRRK2 biology is still poorly understood and these protein clearly are central to the disease etiology: how are they related? Does the spread of pathology relate to normal cell biology or is it simply pathological? These are some of the basic biological questions we need to address. Identifying genes has also given us the opportunity to identify people presymptomatically and should help us identify biomarkers for the disease progression. The last 15 years have brought enormous progress, but there is still much work to be done before we can translate this progress into clinical practice.

## Figures and Tables

**Table 1:** Mendelian Genes that Lead to Parkinsonism and Their Pathology

Locus	Genes	Clinical Features	Pathology
**Dominant**			

PARK1/4	α-Synuclein	Typical PD but can sometimes have a dementia presentation	Lewy bodies
PARK8	LRRK2	Typical PD	Usually Lewy bodies: sometime tangles, sometimes neither
FTDP-17	MAPT	Most mutations have a dementia phenotype but some have a typical PD presentation	Tau/tangle pathology
SCA3	Ataxin3	Usually ataxia in Europeans, but often Parkinsonian especially in Africans	Probably not Lewy bodies. Probably polyglutamine inclusions
SCA2	Ataxin2	Usually ataxia in Europeans, but often Parkinsonian especially in Asians	Probably not Lewy bodies. Probably polyglutamine inclusions

**Recessive**			

PARK2	Parkin	Very slowly progressive early onset disease usually with sleep benefit	Usually not Lewy bodies
PARK6	PINK1	Usually very slowly progressive early onset disease usually with sleep benefit	One case with Lewy bodies
PARK7	DJ-1	Little data, but seems similar to parkin	Not known
PARK9	ATP13A2	Aggressive and complex parkinsonism with many additional features	Not known
PARK14	PLA2G6	Aggressive and complex parkinsonism with many additional features	Lewy bodies
SPG11	Spatacsin	Usually spastic paraplegia but sometimes aggressive and complex parkinsonism with additional features	Not known

**High-Risk Locus**			

Gaucher's (1) locus	GBA	Typical PD	Lewy bodies

**Low-Risk Loci**			

	SNCA	Typical PD	Lewy bodies
	MAPT		Lewy bodies (though tau pathology not systematically assessed
	LRRK2	Typical PD	Lewy bodies
	HLA	Typical PD	Lewy bodies
